# Influence of Zhigancao decoction on chronic heart failure combined with depression

**DOI:** 10.3389/fcvm.2025.1538940

**Published:** 2025-04-14

**Authors:** Ying Wang, Jun Wang, Wang Lv, Hu Chen, Yang Zhang, Qian Yang, Xiaoli Ma, Run Guo, Qianyu Zhang

**Affiliations:** ^1^ Department of Chinese Medicine, Cangzhou Central Hospital, Cangzhou, Hebei, China; ^2^ Department of Office of Academic Research, Cangzhou Central Hospital, Cangzhou, Hebei, China; ^3^ Department of Fifth Cardiology, Cangzhou Central Hospital, Cangzhou, Hebei, China

**Keywords:** heart failure, depression, Chinese traditional medicine, quality of life, combination (combined) therapy

## Abstract

Chronic heart failure (CHF) combined with depression represents a significant clinical challenge due to the mutual exacerbation of physical and psychological symptoms. This study investigated the therapeutic effects of Zhigancao decoction, a traditional Chinese medicine, in combination with conventional Western treatments in patients with CHF and depression. A total of 122 patients were enrolled and divided into two groups: a control group receiving standard Western treatment, and an observation group receiving Zhigancao decoction in addition to conventional therapy. Outcomes were assessed by evaluating the clinical efficacy, cardiac function, inflammatory markers, depressive symptoms, and quality of life. The Zhigancao decoction group exhibited significantly higher efficacy rates, improved left ventricular ejection fraction (LVEF), reduced levels of inflammatory markers [N-terminal pro-brain natriuretic peptide (NT-proBNP), matrix metalloproteinase-9 (MMP-9), and high-sensitivity C-reactive protein (hs-CRP)], and lower scores on depression scales, compared to the control group. Furthermore, the quality of life significantly improved in the Zhigancao decoction group. These findings underscore the potential of Zhigancao decoction as an effective adjunct to conventional treatments for managing CHF combined with depression, offering a holistic approach that integrates physical and mental health improvements.

## 1 Introduction

Heart failure (HF) can occur due to various causes, resulting in abnormal cardiac systolic and/or diastolic function (1). It is characterized by cardiac circulatory disorders and is an advanced stage of cardiovascular disease requiring long-term management (2, 3). Chronic HF (CHF) is marked by persistent symptoms such as dyspnea, fatigue, and fluid retention (4). Western medicine has an effective drug system for CHF that offers significant short-term symptom relief and improves laboratory indicators (5, 6). However, challenges in long-term management contribute to high mortality rates (7). CHF poses serious health risks and often leads to a poor prognosis, causing mental and physical strain that can result in depression (8). Additionally, depression can accelerate CHF progression and negatively affect outcomes, with both conditions acting as risk factors for each other (9).

Although Western medicine has developed a comprehensive drug system to manage CHF, limitations in long-term treatment remain, particularly in addressing the psychological burden of depression (10–12). However, Traditional Chinese medicine (TCM) believes that CHF combined with depression can be attributed to the “heart water” and “depression syndrome,” primarily caused by long-term deficiency of heart and kidney Yang, stagnation of qi activity, and stagnation of liver qi (13). Zhigancao decoction has been used historically to treat cardiovascular diseases, especially arrhythmias, and recent studies have suggested its potential benefits in improving cardiac function and reducing depressive symptoms (14–16). However, there is a paucity of clinical evidence on its efficacy in treating CHF combined with depression, which presents a significant gap in the current literature.

Therefore, in this study, we aimed to investigate the effects of Zhigancao decoction in patients with CHF and depression, focusing on its impact on clinical outcomes, cardiac function, inflammatory markers, and depressive symptoms. Our primary objective was to assess improvements in clinical efficacy and cardiac function. The secondary objective was to evaluate changes in depressive symptoms, quality of life, and levels of inflammation, addressing the need for more comprehensive treatment strategies for this dual condition.

## 2 Methods

### 2.1 Clinical data

The research was ramdomized trial. A total of 122 patients with CHF and depression who were treated at our hospital between March 2020 and October 2022 were chosen for this study. The patients were categorized into two groups with ramdom number table method, with 60 case in the one group and 62 cases in the other group. The 60 patients in the control group (CG) received conventional Western medicine treatment and 62 patients in the observation group (OG) received Zhigancao decoction on the grounds of the CG. No differences were observed with respect to sex, average age between the two groups (*p* > 0.05, Table 1). All patients signed an informed consent form for participating in the study. Moreover, all clinical data and information with confidentiality of patients received safeguarding.

**Table 1:** Patients’ general information in both the groups.

Index	Control group (*n* = 60)	Observation group (*n* = 62)	*p*-value
Sex (male/female)	32/28	34/28	>0.05
Average age (years)	65.06 ± 6.23	65.11 ± 6.21	>0.05
NYHA grade (II/III/IV)	18/22/20	16/25/21	>0.05
Duration of heart failure	12.32 ± 3.10	12.10 ± 3.20	>0.05
Duration of depression	3.16 ± 0.92	3.45 ± 1.00	>0.05
Depression severity (mild/moderate/severe)	37/17/6	34/20/8	>0.05

### 2.2 Diagnostic criteria

Diagnostic criteria for CHF: Patients with CHF who met the Western medical diagnostic criteria of CHF and grades III–IV cardiac function.

Diagnostic criteria for depression: Evaluation using patient health questionnaire-9 (PHQ-9) screening scale and self-rating depression scale (SDS). The total PHQ-9 score is 27 points: 0–4 points indicates no depression, 5–9 points implies mild depression, 10–14 points suggests moderate depression, 15–19 points indicates moderately severe depression, and ≥20 points indicates severe depression. The cutoff value of the SDS standard score was 53 points; 53–62 is classified as mild depression, 63–72 as moderate depression, and >72 as severe depression.

TCM syndrome criteria: Diagnostic criteria based on the Guiding Principles for Clinical Research of New Chinese Medicine released by the State Administration of Technical Supervision Syndrome of liver depression, qi deficiency, and blood stasis: chest tightness and shortness of breath, palpitations, fatigue, self-sweating, especially movement, white or dark red face, cyanosis of the lips, exposure of the jugular veins, and accumulation of blocks under the hypogastrium. The tongue turns dark purple or ecchymotic and the pulse is heavy and thin, with astringent or knot generation.

### 2.3 Inclusion criteria

(1) Aged 18–80 years. (2) Patients with CHF who met the diagnostic criteria for CHF and cardiac function ranging from grade Ⅲ to Ⅳ. (3) Use PHQ-9 and SDS to value the diagnosis of depression. (4) TCM syndrome differentiation based on liver depression, qi deficiency, and blood stasis.

### 2.4 Exclusion criteria

(1) Incomplete medical records. (2) Allergic to therapeutic drugs. (3) Pregnant and lactating women.

### 2.5 Methods

Patients in the CG were administered Western medicine according to the 2014 Guidelines for the Diagnosis and Treatment of Heart Failure in China. Diuretics (furosemide tablets 20 mg qd; Shanghai Zhaohui Pharmaceutical Co., LTD., Shanghai, China), angiotensin-converting enzyme inhibitor (ACEI, enalapril maleate tablets, 10 mg qd; Shi Yao Group European Pharmaceutical Co., LTD.,), *β*-blockers [bisoprolol fumarate tablets, 5 mg qd; Merck Pharmaceutical (Jiangsu) Co., LTD.], and aldosterone antagonists (spironolactone tablets, 20 mg qd; Suzhou Hongsen Pharmaceutical Co., LTD.) were used as basic treatment. The course of treatment was 2 months.

Patients in the OG group received Zhigancao decoction based on the CG. Medicine composition: Radix glycyrrhizae preparata 12 g, ginseng 6 g, ginger 9 g, cassia twig 9 g, raw Rehmannia 50 g, Colla corii asini 6 g, hemp seed 10 g, jujube 10 g, calcinated keelbone 15 g, and Radix ophiopogonis 10 g. One dose per day, divided into 2 doses. The course of treatment was 2 months.

### 2.6 Observation indicators

(1) The efficacy was evaluated according to SDS and TCM syndrome scores before and after treatment. Obvious effects: SDS and TCM syndrome scores decreased by ≥75% after treatment. Effective: SDS and TCM syndrome scores reduced by 50%–75% after treatment. Ineffective: SDS and TCM syndrome scores decreased by <50% after treatment.
(2) The scores of the patients’ symptoms and signs were evaluated using the Guiding Principles of Clinical Research on New Chinese Medicine.
(3) Fasting peripheral venous blood (3 ml) was collected. Serum N-terminal pro-B-type natriuretic peptide (NT-proBNP) levels were measured using electrochemiluminescence immunoassay, and high-sensitivity C-reactive protein (hs-CRP) along with matrix metalloproteinase-9 (MMP-9) levels were examined using enzyme-linked immunosorbent assay.
(4) Left ventricular ejection fraction (LVEF) was examined using echocardiography to assess cardiac function.
(5) PHQ-9 and SDS were used to evaluate the patients’ depression (17).
(6) Minnesota Living with Heart Failure (MLHF) questionnaire was used to evaluate the patients’ quality of life (18). The score ranged from 0 to 105, with a higher score indicating a lower quality of life.

### 2.7 Statistical analysis

Adapt SPSS 16.0 (International Business Machines Corporation, USA) was used to analyze the data. Measurement data are exhibited as mean ± standard deviation (*x* ± *s*), and the *T* test was used for comparison. Counting data are exhibited as rate (%) and the *χ*^2^ test was applied for comparison. Statistical significance was set at *p* < 0.05.

## 3 Results

### 3.1 Zhigancao decoction elevates total effective rate for patients in CHF patients with depression

**Figure 1:**
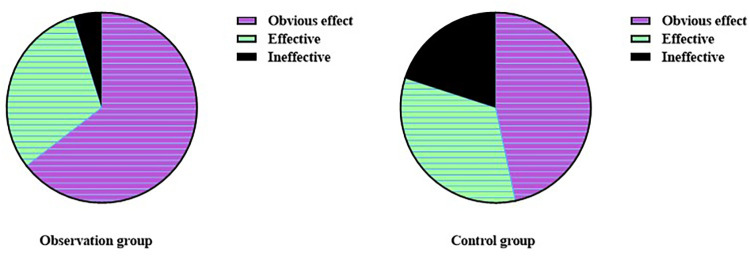
Total effective rate in both the groups.

The total effective rate in OG was 95.16%, which was higher than the rate of 80.00% in CG (*χ*^2^ = 6.50, *p* < 0.05, Figure 1), suggesting that Zhigancao decoction is effective in treating CHF combined with depression.

### 3.2 Zhigancao decoction ameliorates scores of TCM symptoms and signs in CHF patients with depression

**Figure 2:**
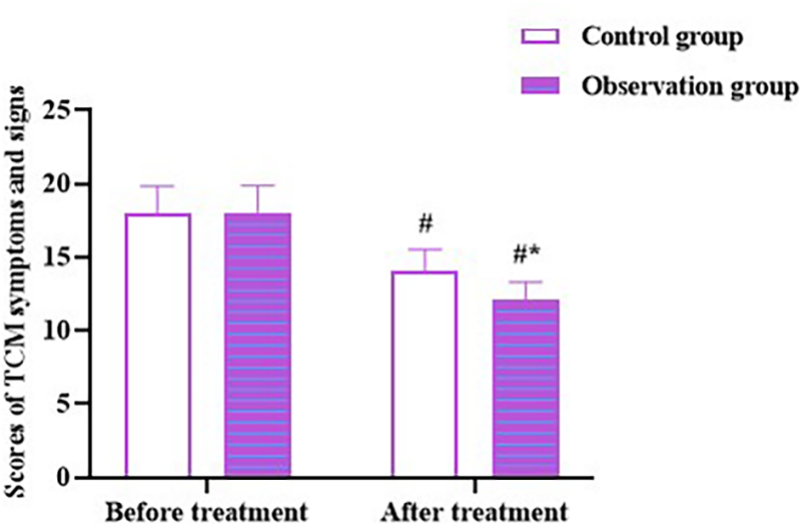
TCM symptoms and signs scores in both the groups. Compared to before treatment, ^#^*p* < 0.05. Compared to CG, *p < 0.05. TCM: Traditional Chinese medicine.

No differences were observed in the symptoms and signs scores of TCM between the two groups before therapy (*p* > 0.05). The scores declined in both the groups after therapy (*p* < 0.05), and scores in the OG were lower than those in the CG (*p* < 0.05; Figure 2). These outcomes clarify that Zhigancao decoction could improve TCM symptoms and signs in patients with CHF combined with depression.

### 3.3 Zhigancao decoction leads to NT-proBNP level reduction in CHF patients with depression

**Figure 3:**
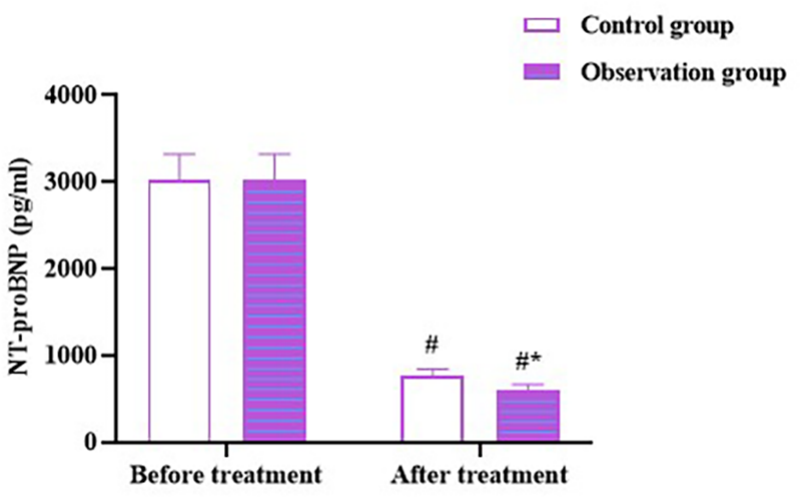
NT-proBNP level in both the groups. Compared to before treatment, ^#^*p* < 0.05. Compared to CG, *p < 0.05. NT-proBNP: N-terminal pro-B-type natriuretic peptide.

No difference was observed in the NT-proBNP levels between the two groups before therapy (*p* > 0.05). After therapy, the NT-proBNP level in both groups decreased (*p* < 0.05), and the level in the OG was lower than that in the CG (*p* < 0.05; Figure 3). These results suggest that Zhigancao decoction can improve HF in patients with CHF and depression.

### 3.4 Zhigancao decoction attenuates levels of inflammatory cytokines in CHF patients with depression

**Figure 4:**
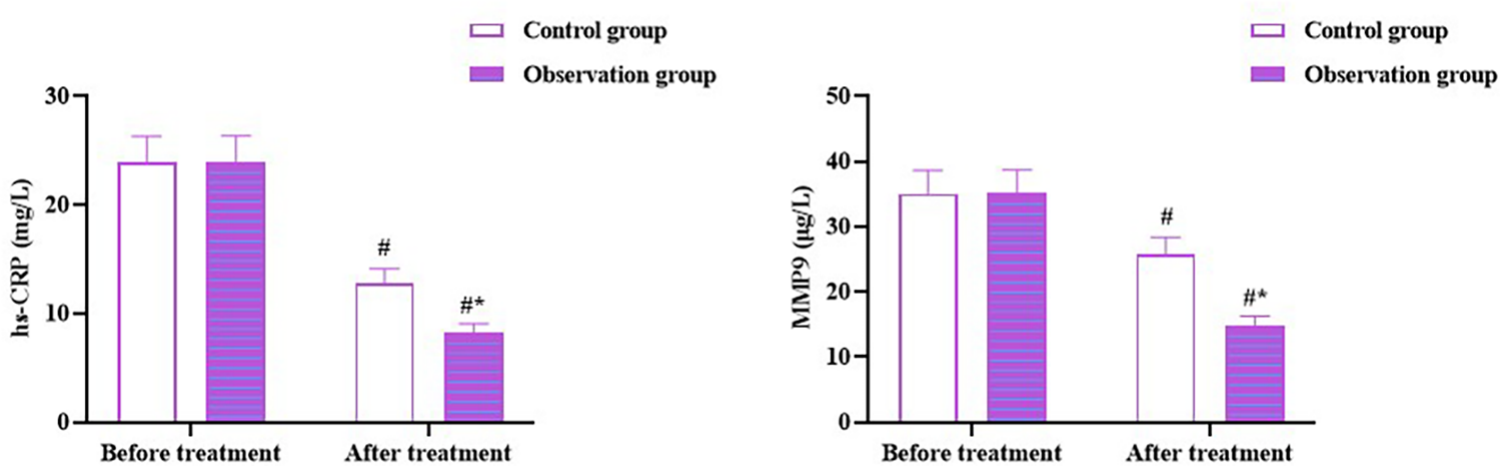
Levels of inflammatory cytokines in both the groups. Compared to before treatment, ^#^*p* < 0.05. Compared to CG, *p < 0.05. hs-CRP: high-sensitivity C-reactive protein; MMP-9: matrix metalloproteinase-9.

No significant difference was observed in MMP-9 and hs-CRP levels between the two groups before therapy (*p* > 0.05). MMP-9 and hs-CRP levels in both the groups reduced after therapy (*p* < 0.05), and the levels in the OG were lower than those in the CG (*p* < 0.05; Figure 4). These outcomes indicate that Zhigancao decoction can reduce inflammatory response in patients with CHF combined with depression.

### 3.5 Zhigancao decoction enhances LVEF in CHF patients with depression

**Figure 5:**
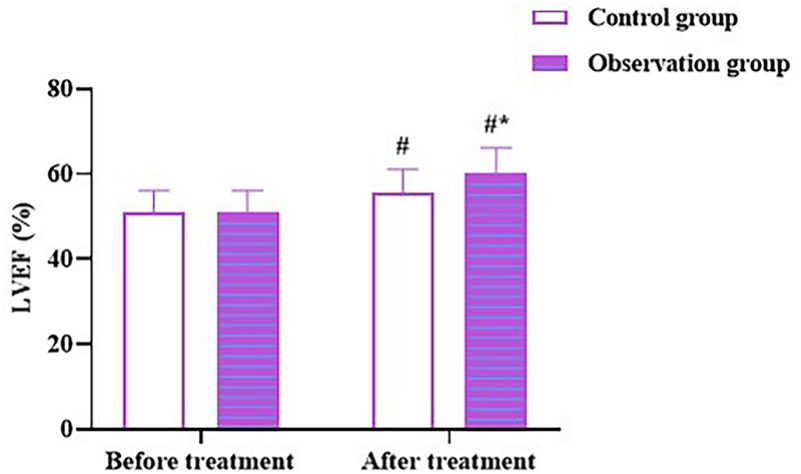
LVEF in both the groups. Compared to before treatment, ^#^*p* < 0.05. Compared to CG, *p < 0.05. LVEF: left ventricular ejection fraction.

No difference was observed in LVEF between the two groups before therapy (*p* > 0.05). LVEF levels in both the groups was elevated after therapy (*p* < 0.05), and levels in the OG were higher than those in the CG (*p* < 0.05; Figure 5). All outcomes indicate that Zhigancao decoction could improve cardiac function in patients with CHF and depression.

### 3.6 Zhigancao decoction results in deline in PHQ-9 and SDS scores in CHF patients with depression

**Figure 6:**
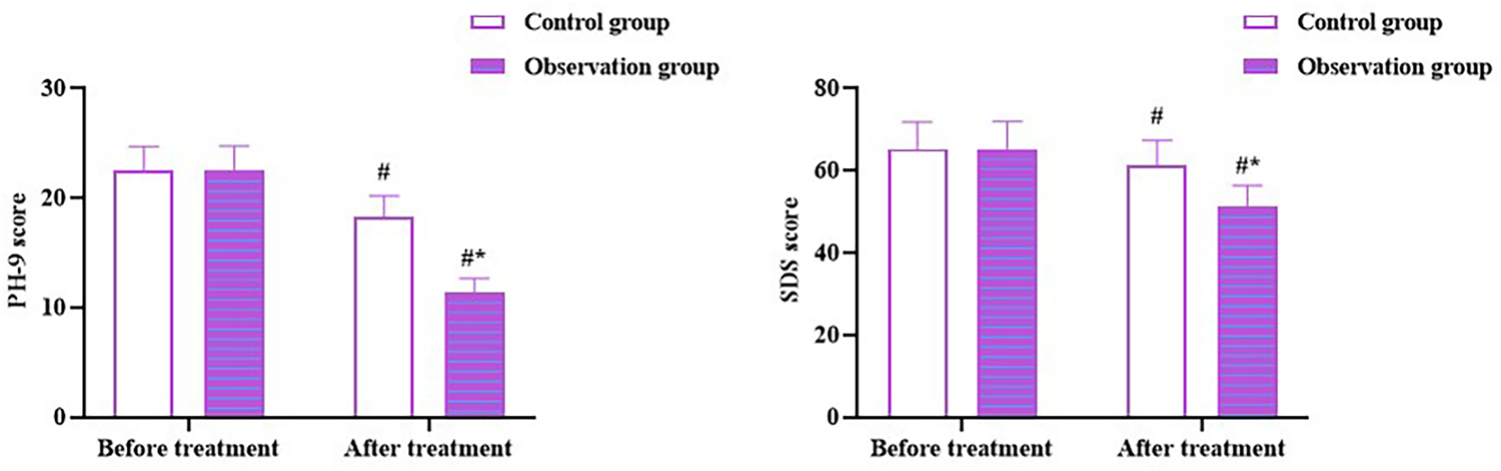
PHQ-9 and SDS scores in both the groups. Compared to before treatment, ^#^*p* < 0.05. compared to CG, *p < 0.05. SDS: self-rating depression scale; PHQ-9: patient health questionnaire-9.

No differences were observed in PHQ-9 and SDS scores between the two groups before therapy (*p* > 0.05). After therapy, the PHQ-9 and SDS scores declined in both the groups (*p* < 0.05), and scores in the OG were lower than those in the CG (*p* < 0.05; Figure 6). All these outcomes indicate that Zhigancao decoction could improve the depressive state of patients with CHF and depression.

### 3.7 Zhigancao decoction improves quality of life in CHF patients with depression

**Figure 7:**
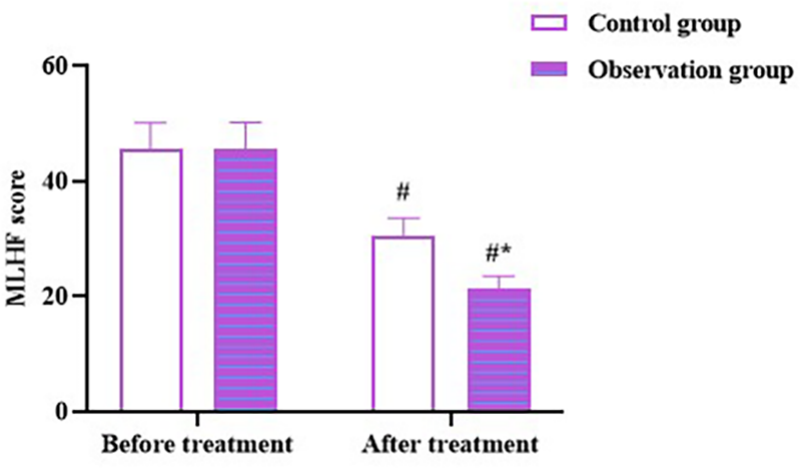
Life quality of patients in both the groups. Compared to before treatment, ^#^*P* < 0.05. compared to CG, *P < 0.05. MLHF: Minnesota living with heart failure.

No difference was observed in the MLHF scores between the two groups before therapy (*p* > 0.05). After treatment, the MLHF score decreased in both the groups (*p* < 0.05), and scores in the OG was lower than those in the CG (*p* < 0.05; Figure 7). All these outcomes indicate that Zhigancao decoction could promote the quality of life of depressed patients with CHF.

## 4 Discussion

CHF is the final pathological process in many cardiovascular diseases (19). Because the patient is in a chronic disease state, the course of the disease is repeated, the quality of life is reduced, and the economic burden is substantial; furthermore, chronic conditions are often complicated by depression, and poor heart function of the patient is accompanied by a higher probability of depression (20). Due to poor compliance with treatment recommendations, poor cardiac autonomic nerve function, aggravated inflammatory processes, thrombosis, and endothelial dysfunction, depression further worsens cardiac function and increases mortality in patients with CHF (21).

Currently, therapies for CHF complicated by depression mainly include drug therapy, psychological intervention, social support therapy, and exercise therapy. Tricyclic antidepressants (TCAs) and selective serotonin reuptake inhibitors (SSRIs) are commonly used antidepressants, although their effects are unclear (22). However, TCAs have adverse cardiovascular side effects, including increased heart rate, induction of orthostatic hypotension, and slow ventricular conduction (23). One of the pharmacological characteristics of TCAs is their similarity to class I antiarrhythmic effects, which can block the sodium and potassium channels of the myocardial cell membrane, thus affecting the process of cardiomyocyte depolarization and repolarization (24). Moreover, studies have confirmed that TCAs have the risk of prolonging the QT interval and stimulating torsades de pointes (25). It also increases the risk of sudden cardiac death. In addition, some antidepressants have a long course of treatment, slow onset, drug resistance, high prices, and many adverse reactions; therefore, their clinical application is limited (26).

TCM believes that CHF belongs to the terminal stage of various cardiovascular diseases (27). The initial pathological development is mostly due to weakness of the heart and qi, and lack of blood circulation, resulting in decreased blood flow and blood stasis (28). TCM believes that the heart is the main blood vessel and is associated with consciousness, while the liver stores blood and regulates emotions. CHF leads to emotional depression, liver qi stagnation with multiple consequences, and poor qi (29). Therefore, the clinical treatment of CHF combined with depression benefits qi, promotes blood circulation, and relieves liver depression.

In the Zhigancao decoction recipe, raw Rehmannia is the monarch drug that nourishes Yin and blood (30). Simultaneously, Radix glycyrrhizae preparata, ginseng, Colla corii asini, Radix ophiopogonis, hemp seed, and jujube are ministerial drugs. The combined use of Glycyrrhizae preparata, ginseng, and jujube can benefit the heart and qi, and invigorate the spleen (31–33). The combination of Colla corii asini, Radix ophiopogonis, and hemp seed can nourish the heart Yin, and the heart and blood (34–36). Cassia twigs and ginger as the adjuvant, have the effect of warm heart Yang and promote blood circulation (37, 38). The drugs used together can benefit qi and nourish yin, and improve qi and blood, which can improve the depressive symptoms of patients with CHF (39).

LVEF is commonly used in clinical evaluation of cardiac function; LVEF is often abnormally decreased in patients with refractory HF, affected by vasospasm, increased myocardial load, and other factors (40). NT-proBNP is a marker of HF, and when the ventricle is stretched, changes in its tension can increase the release of NT-proBNP (41). MMP-9 is widely present in the myocardial tissue, and left ventricular cardiomyocytes may be the main source of MMPs in myocardial tissue (42). The expression level of MMP-9 is significantly increased owing to structural and compositional changes in the left ventricle of patients with CHF (43). hs-CRP is a non-specific inflammatory protein commonly used in clinical practice, and its levels can reflect the instability of vascular endothelial function (44).

In this study, the outcomes demonstrated that after therapy, the total effective rate for patients in the OG was elevated relative to that for patients in the CG; TCM symptoms and signs scores in OG were lower relative to those in CG; NT-proBNP, hs-CRP, and MMP-9 levels in OG were reduced relative to CG; PHQ-9 and SDS scores declined in OG relative to CG, LVEF level was higher in OG compared to CG, and MLHF score was lower in OG relative to CG. These data suggest that Zhigancao decoction could improve the clinical efficacy of patients with CHF combined with depression, reduce TCM symptoms and signs, promote cardiac function, reduce inflammation, relieve depression, and improve the quality of life. Consistently, it has been reported that Zhigancao decoction plus conventional medicine demonstrate good efficacy and fewer adverse reactions than conventional medicine in the treatment of arrhythmia (45). Yang et al. discovered that the combination of Chinese herbal medicines and conventional medical treatment increased the effective rate of cardiac function, reduced depressive status, and promoted the quality of life in patients with CHF complicated by depression (46).

There still exist some limitations in our research design. The key issues included a small sample size (122 patients) and a short intervention period (2 months), which may limit generalizability and obscure long-term effects. Additionally, reliance on subjective scales (e.g., PHQ-9, SDS) for depression assessment introduced potential bias. The abstract/introduction section lacked clarity on study design (e.g., whether it was randomized or observational) and deeper context on the shortcomings of current Western therapies for CHF with depression. In the future, improvements can be made to address this problem, such as a deeper analysis on key roles of individual herbal components (e.g., glycnsisitin A, one of the bicyclic peptides, as bioactive molecule of Zhigancao decoction for the treatment of CHF with depression) and potential placebo effects, especially given subjective outcome measures, to further evaluate the clinical efficacy of Zhigancao decoction and explore its underlying therapeutic mechanism, which may provide a theoretical basis for the treatment of CHF complicated with depression using TCM.

## 5 Conclusion

Based on Western medicine treatment, Zhigancao decoction can relieve the clinical symptoms and observation indicators of patients with CHF complicated by depression, which is valuable for clinical use. Nevertheless, larger randomized trials, extended follow-ups, and mechanistic studies are still needed for validating and expanding on our findings.

## Data Availability

The original contributions presented in the study are included in the article/Supplementary Material, further inquiries can be directed to the corresponding author.
